# Supplementary material for: Longitudinal evaluation of the impact of traditional rainbow trout farming on receiving water quality in Ireland
Source: PeerJ. 2018 Jul 24;6:e5281. doi: 10.7717/peerj.5281 (PMC6063216; doi:10.7717/peerj.5281)
Supplement: Table S1 [file peerj-06-5281-s002.docx]

| **Farm** | **TON** | **NO_2_-N** | **NH_4_-N** | **BOD_5_** | **TSS** | **Turb.** | **PO_4_-P** | **DO** | **pH** | **Temp.** | **Total** |
| --- | --- | --- | --- | --- | --- | --- | --- | --- | --- | --- | --- |
| **1** | 16 | 17 | 17 | 17 | 17 | 17 | 17 | 17 | 17 | 17 | 169 |
| **2** | 29 | 30 | 30 | 30 | 30 | 22 | 30 | 16 | 30 | 25 | 272 |
| **3** | 41 | 41 | 42 | 42 | 41 | 31 | 42 | 35 | 42 | 40 | 397 |
| **4** | 40 | 40 | 41 | 41 | 41 | 31 | 41 | 34 | 41 | 38 | 388 |
| **Total** | 126 | 128 | 130 | 130 | 129 | 101 | 130 | 102 | 130 | 120 | 1226 |
