# Supplementary material for: Longitudinal evaluation of the impact of traditional rainbow trout farming on receiving water quality in Ireland
Source: PeerJ. 2018 Jul 24;6:e5281. doi: 10.7717/peerj.5281 (PMC6063216; doi:10.7717/peerj.5281)
Supplement: Supplementary file 3 [file peerj-06-5281-s003.docx]

| | January | | | | February | | | | March | | | | April | | | | May | | | | June | | | | July | | | | August | | | | September | | | | October | | | | November | | | | December | | | |
|---|---|---|---|---|---|---|---|---|---|---|---|---|---|---|---|---|---|---|---|---|---|---|---|---|---|---|---|---|---|---|---|---|---|---|---|---|---|---|---|---|---|---|---|---|---|---|---|---|
| | **1** | **2** | **3** | **4** | **1** | **2** | **3** | **4** | **1** | **2** | **3** | **4** | **1** | **2** | **3** | **4** | **1** | **2** | **3** | **4** | **1** | **2** | **3** | **4** | **1** | **2** | **3** | **4** | **1** | **2** | **3** | **4** | **1** | **2** | **3** | **4** | **1** | **2** | **3** | **4** | **1** | **2** | **3** | **4** | **1** | **2** | **3** | **4** |
| **2005** | | | | | | | | | | | | | | | | | | | | | | | | | | | 3 | 4 | | 2 | 3 | 4 | | | | | | | | | | | | | | | | |
| **2006** | | | | | | | | | | | | | | | | | | | | | | | | | | 2 | 3 | 4 | | | | | | | | | | | | | | 2 | 3 | 4 | | | | |
| **2007** | | | | | | | | | | | | | | 2 | 3 | 4 | | | | | | | | | | 2 | 3 | 4 | | 2 | 3 | 4 | | | | | | 2 | 3 | 4 | | | | | | | | |
| **2008** | | | | | | | 3 | 4 | 1 | 2 | 3 | 4 | | | | | | | 3 | 4 | 1 | 2 | 3 | 4 | | | | | | | 3 | 4 | 1 | 2 | 3 | 4 | | | | | | | 3 | 4 | 1 | 2 | 3 | 4 |
| **2009** | | | | | | | 3 | 4 | 1 | 2 | 3 | 4 | | | | | | | 3 | 4 | 1 | 2 | 3 | 4 | | | | | | | | | 1 | 2 | 3 | 4 | | | | | | | 3 | 4 | | | | |
| **2010** | | | | | | | | | | | | | | | 3 | 4 | | | | | | | | | | | | | | | | | | | | | | | 3 | | | | | | | | | |
| **2011** | 1 | | 3 | 4 | | | | | | | | | 1 | | 3 | 4 | | | | | | | | | 1 | 2 | 3 | 4 | | | | | | | | | 1 | 2 | 3 | 4 | | | 3 | 4 | | | | |
| **2012** | 1 | 2 | 3 | 4 | | 2 | 3 | 4 | | | | | 1 | 2 | 3 | 4 | | | | | | | | | 1 | 2 | 3 | 4 | | | | | | | | | | | | | 1 | 2 | 3 | 4 | | | | |
| **2013** | 1 | 2 | 3 | 4 | | | | | | | | | 1 | 2 | 3 | 4 | | | | | | 2 | 3 | 4 | | | | | | 2 | 3 | 4 | | | | | 1 | 2 | 3 | 4 | | | | | | | | |
| **2014** | | | | | | | | | | | | | | 2 | 3 | 4 | | | | | | | | | | | | | | | | | | | | | | 2 | 3 | 4 | | | | | | | | |
| **2015** | | | | | | 2 | 3 | 4 | | | | | | | | | | | | | | | | | | | | | | | | | | | | | | | | | | | | | | | | |
